# News and Coming Events

**Published:** 1908-04-18

**Authors:** 


### NEWS AND COMING EVENTS.

The Lord Mayor of London has decided to give a Mansion House dinner on May 27 with a view to collecting money for the London Hospital.

The Gresham Lectures on Physic, founded by Sir Thomas Gresham, will be read to the public gratuitously at 6 p.m. in the theatre of Gresham College, Basinghall Street, by Dr. Sandwith, on May 5, 6, 7, and 8.

The Jacksonian prize of the Royal College of Surgeons for the year 1907 was not awarded. The subject for the prize for the year 1909 will be: The Treatment of Surgical Affections by Vaccines and Anti-toxins.

At the spring graduation ceremony of the University of Edinburgh, honorary degrees of doctor of laws were conferred on Dr. James Affleck, consulting physician to the Edinburgh Royal Infirmary, and Dr. Richard Caton, the Lord Mayor of Liverpool.

Mr. T. J. Walker, M.R.C.S., consulting surgeon to the Peterborough Infirmary, and Mr. H. P. Keatinge, M.R.C.S., director of the Egyptian Government Medical School at Cairo, have been elected Fellows of the Royal College of Surgeons of England.

The First Lord of the Admiralty has appointed Inspector-General James Porter, C.B., M.D., to be Director-General of the Medical Department of the Royal Navy, in succession to Inspector-General Sir Herbert M. Ellis, K.C.B., F.R.C.S., LL.D., who has resigned.

Mr. J. Ward Cousins and Mr. Henry Rundle, at the conclusion of forty-eight years and thirty-four years' service respectively, have resigned their posts on the surgical staff of the Royal Portsmouth Hospital, and have been elected honorary consulting surgeons to that institution.

Sixteenth International Medical Congress.—Professor Emil Grosz, of Budapest, secretary-general of the Sixteenth International Medical Congress, which is to be held in Budapest, August 29 to September 4, 1909, has sent out the preliminary programme of the Congress. There will be twenty-one sections. The secretarial office of the congress is Esterházy-Utcza, 7 Budapest VIII, Hungary.

The Council of the Incorporated Institute of Hygiene are promoting a special exhibition, illustrating the requirements of infant life and the care and health of children, which will be opened June 15, 1908. This exhibition is intended to demonstrate the progress of recent years in providing for the welfare, comfort and training of the child, and it is proposed to exhibit the latest improvements connected with foods, clothing, the nursery, the bedroom, recreation, physical exercise, school life and education.

Dr. Oswald Auchinleck Browne, assistant registrar of the Royal College of Physicians and consulting physician to the Metropolitan Hospital, died on April 9 of heart failure after an operation. Dr. Oswald Browne studied at Cambridge University and St. Bartholomew's Hospital, and qualified in 1881. He took the M.D. degree in 1897, and became a Fellow of the Royal College of Physicians in 1898. He was physician to the Royal Hospital for Diseases of the chest and to the Alexandra Hospital for Hip Disease.

The Royal Hungarian Minister of the Interior offers the sum of 1,000 crowns as a prize to the author of the best work on the Ætiology of Trachoma. All essays, whether in manuscript or published for the first time in 1907 or 1908, will be eligible for the competition, provided they are in English, Hungarian, French or German, and that they are received on or before December 31, 1908, by the Minister of the Interior. They should be addressed "Belügyministerium, Budapest, Hungary." The winner will be announced at the inaugural session of the sixteenth International Medical Congress at Budapest in 1909.

Sir Shirley Murphy presided at a recent meeting of the Incorporated Society of Medical Officers of Health, at Russell Square, at which Mr. Francis E. Fremantle read a paper entitled "The Garden City, Rural Housing, and the Government." Mr. Fremantle severely criticised Mr. Burns's Town Planning Bill. Amongst other shortcomings, it does nothing to remedy the anomalies of the position of district medical officers of health and sanitary inspectors. He is of opinion that it is a reactionary measure, framed on wrong lines, and hardly capable of proper amendment. A discussion followed, and it was stated that the Council of the Society is specially considering the Bill in question, with a view to securing the permanency of the appointment of medical officers of health. Mr. Edward Sergeant (Medical Officer of Health for the County of Lancaster) has been elected President of the Society for the year 1908-1909.

The President and Vice-Presidents of the Royal College of Surgeons of England, in pursuance of a resolution adopted by the Council, have addressed a letter to Sir Henry R. Swanzy, President of the Royal College of Surgeons in Ireland, expressing the strong sense which the Council entertain as to the importance of maintaining in Ireland a Royal College of Surgeons, and the hope that in any Bill which is brought forward to establish a new Irish University provision may be made to safeguard the interests of the Irish College of Surgeons. The letter paid tribute to the important services to surgical education rendered by the College through its school and examinations, and expressed the opinion that it would be a misfortune, not only to the profession, but to the public, if legislation were introduced which should result in diminishing or impairing the functions now exercised by the Royal College of Surgeons in Ireland.

The Dean of Westminster has given his consent for a service for members of the University of London, to be held in Westminster Abbey on Presentation Day, Wednesday, May 6, at 6 p.m. With the consent of the Dean, the Bishop of Birmingham has accepted the invitation to preach the sermon. The service will be open to all persons officially connected with the University of London as teachers or otherwise, to all graduates and undergraduates, and all regular students of schools of the University. Full academic dress will be worn. Tickets admitting to the reserved space in the Abbey will be sent to all persons eligible who apply for them to Mr. L. S. Kempthorne, University of London, University College, Gower Street, W.C., enclosing a stamped addressed envelope. Applicants should state whether they are graduates or undergraduates. Application for tickets should be made as soon as possible, and not later than April 29. It is particularly requested that applications for tickets should on no account be addressed to the Dean or to any other official of the Abbey.

Dr. G. Harrison Orton has been appointed Medical Officer in Charge of the $x$-Ray Department at St. Mary's Hospital.

Lord Ancaster, President of the Evelina Hospital for Sick Children, Southwark, will take the chair at the annual Court of Governors on April 22.

At University College, Bristol, Dr. J. M. Fortescue Brickdale, M.A., M.D., B.Ch., Oxon., has been appointed director of the public health laboratory of the College.

At a meeting of the Royal College of Physicians of London held on April 13, Sir Richard Douglas-Powell, M.D., was, by a practically unanimous vote, re-elected President of the College.

On April 13 a daring burglary was perpetrated at the Western Fever Hospital of the Metropolitan Asylums Board in Seagrove Road, Fulham. According to the published accounts, a safe was forced open with a jemmy, and notes, gold, and silver stolen to the value of £155.

On April 6 Sir Dyce Duckworth, M.D., LL.D., visited the Royal Hospital, Haslar, to distribute the prizes won by recently trained surgeons, R.N., at the termination of the course of instruction. Sir Herbert Ellis, K.C.B., the Medical Director-General of the Navy, was also present. The prizes were awarded as follows: The gold medal for hygiene, etc., to Surgeon G. B. Scott, of St. Bartholomew's Hospital. The silver medal and books for practical analysis, etc., to Surgeon J. McCutcheon, of Edinburgh University. The microscope for tropical medicine, etc., to Surgeon G. Carlisle, of Guy's Hospital.

The seventy-sixth annual meeting of the British Medical Association will be held at Sheffield in July 1908. The President's address will be delivered on Tuesday, July 28, in the Firth Hall of the University, and the sections will meet on the three following days. The annual representative meeting will begin at the close of the previous week, probably on Friday, July 24. The President-elect is Mr. Simeon Snell, F.R.C.S.Edin., ophthalmic surgeon, Royal Infirmary, Sheffield, and the Hon. Local Secretary Mr. Sinclair White, M.Ch., F.R.C.S., Ranmoor, Sheffield. The address in medicine will be delivered by Dr. Kingston Fowler, the address in surgery will be delivered by Mr. R. J. Pye-Smith, F.R.C.S.Eng., and the popular lecture, on "Dust and Disease," will be delivered by Mr. Edmund Owen, LL.D., F.R.C.S.Eng.

The annual meeting of the Hospital Saturday Fund was held on April 11 at the Mansion House, under the presidency of the Lord Mayor. Canon Fleming presented a report of the work for 1907 on behalf of Sir Savile Crossley (chairman of the Fund), who was prevented from attending. The income of the Fund last year was £27,140, an increase of £680 on the amount collected in 1906. The debt on the freehold premises has now been wiped off. Mr. N. H. Hamilton Hoare, owing to the state of his health, has retired from the hon. treasurership, and the Hon. H. L. W. Lawson will succeed him in that position. Mr. Sheriff Wakefield moved a resolution approving the principle of collecting small contributions weekly in aid of the metropolitan hospitals and kindred institutions. The Mayor of Southwark seconded the resolution, which was carried unanimously. Mr. Thomas Bevan, Chairman of Council, stated that so far this year the collections have realised £5,000 against £4,400 at the corresponding date last year.

Mr. W. Pearson has been appointed to succeed Mr. A. E. Thomas as Secretary to the Gravesend Hospital, Kent.

Mr. Victor Williamson presided at the annual meeting of the governors and subscribers of the Hospital for Women, Soho Square. During 1907 the number of in-patients was 924, which is larger than in any previous year since the foundation of the institution. There were 3,700 out-patients, who made a total of 14,927 attendances. The expenditure exceeded the income by £20. A festival dinner in aid of the rebuilding fund will be held on May 11. The plans for re-modelling the hospital have been prepared and are being considered by the Committee of King Edward's Fund.

The annual meeting of the Medical Missionary Association was held on April 13 at the Holborn Restaurant, Mr. A. Pearce Gould presiding. Dr. Henry Soltau, the General Secretary and Superintendent, presented the annual report. In this the committee recorded with satisfaction the endorsement by last year's Centenary Conference in China of the principles for which the Association had long contended. Everthing possible should now be done to reinforce mission hospital staffs, in many cases undermanned, and also to equip new centres of work. The Student Volunteer Missionary Conference in Liverpool last January, with its attendance of 2,000, including a good number of medical students, gave ground for hope that before long the men and women required would be forthcoming. Two doctors had been sent out to strengthen the staff of the Union Medical College in Pekin, and a joint committee had been formed representing this Association and the London Missionary Society.

